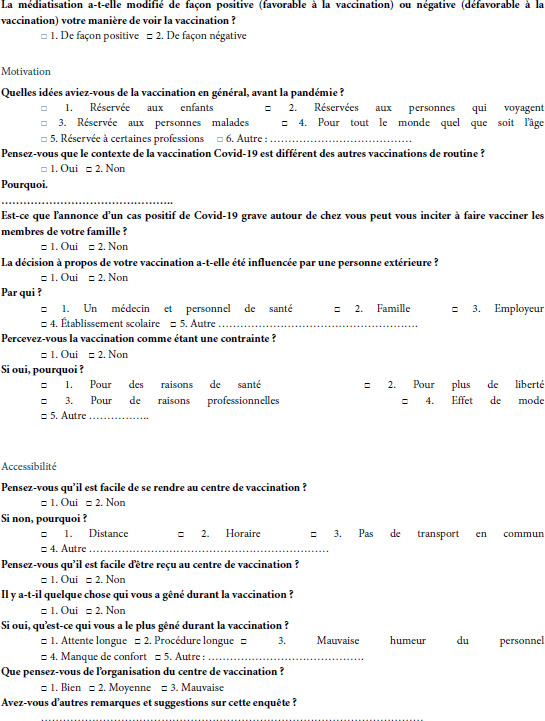# Motivation et perception de l’accessibilité au vaccin par des personnes vaccinées contre la Covid-19 à Makokou au Gabon en 2021–2022

**DOI:** 10.48327/mtsi.v6i1.2026.514

**Published:** 2026-01-29

**Authors:** UlrickJolhy BISVIGOU, Euloge IBINGA, Cédric Noël MINKO, Sydney MAGHENDJI NZONDO, NGOUNGOU EdgardBrice

**Affiliations:** 1 Département d’épidémiologie, biostatistiques et informatique médicale/santé publique, médecine du travail et médecine légale, Faculté de médecine, Université des sciences de la santé, BP 4009, Libreville, Gabon; 2 Unité de recherche en épidémiologie des maladies chroniques et santé environnement, Faculté de médecine, Université des sciences de la santé, BP 11587, Libreville, Gabon; 3 Centre hospitalier régional Bongo Ondimba de Makokou, Gabon

**Keywords:** Connaissances attitudes et pratiques, Covid-19, Motivation, Personnels de santé, Forces de défense et sécurité, Centre de vaccination, Makokou, Gabon, Afrique subsaharienne, Knowledge, Attitudes and Practices, COVID-19, Motivation, Healthcare Workers, Defense and Security Forces, Vaccination Center, Makokou, Gabon, Sub-Saharan Africa

## Abstract

**Introduction:**

La vaccination a été un des moyens de riposte à la pandémie de Covid-19. Elle a été à l’origine de controverses, hésitations et infodémies dans le monde. Le Gabon s’est lancé dans la vaccination de sa population au mois de juillet 2021. Notre étude a pour objectif de décrire les motivations des volontaires, leurs connaissances et leurs niveaux d’acceptation.

**Méthodes:**

Une étude transversale descriptive, de type « Connaissances, attitudes et pratiques », a été menée de mai 2021 à janvier 2022 (8 mois), au sein de l’unique centre de vaccination contre la Covid-19 de Makokou, région sanitaire Est du Gabon. Les volontaires étaient inclus durant leur parcours de vaccination, répondant à un questionnaire lors d’une interview en face à face. Ont été étudiés le profil sociodémographique, les connaissances de la maladie et du vaccin, la perception et le vécu de l’accessibilité.

**Résultats:**

Au total, 303 personnes de 18 à 71 ans ont été incluses. Les sujets âgés de 30 à 40 ans représentaient 39,6 % (n = 120) des sujets inclus. Le sex-ratio était de 2,7. La proportion des participants de nationalité gabonaise était de 86,7 %, la durée médiane du séjour des participants dans la ville de Makokou était de 6 ans (IIQ : 2-15). Les professions les plus représentées étaient le personnel de santé 17,8 % (n = 54), suivies des forces de défense et de sécurité 15,8 % (n = 48), cibles principales du programme. La proportion des participants qui se déclaraient influencés dans leur décision de se faire vacciner était de 42,5 % (n = 129). La proportion de ceux qui pensaient que la vaccination était une contrainte était de 72,6 % (n = 220), pour des raisons de santé 58,2 % (n = 128) ou pour plus de liberté de mouvement 49,5 % (n = 109). Les principales sources d’information étaient les médias 78,2 % (n = 237) et le personnel médical 55,4 % (n = 168). La motivation était associée à la contrainte (OR = 2,2), à la connaissance de certains signes (OR = 0,4), à la perception de la maladie comme étant grave (OR = 0,2) et à l’influence positive des médias (OR = 0,4); l’accessibilité associée à une infection antérieure (OR = 11) ou à une contrainte de l’employeur (OR = 0,2).

**Conclusion:**

La décision de se faire vacciner contre la Covid-19 a été beaucoup influencée par les médias et les contraintes socioprofessionnelles. Devant un accès au vaccin considéré comme facile, la motivation aurait dû être la recherche d’un meilleur état de santé.

## Abstract (en)

**Introduction:**

Vaccination is one of the responses to the ongoing global COVID-19 pandemic. However, it has also sparked controversy, hesitation, and infodemics worldwide. Gabon began vaccinating its population in July 2021. Our study aims to describe the motivations, knowledge, and levels of acceptance of volunteers.

**Methods:**

We conducted a descriptive, cross-sectional, "knowledge, attitudes, and practices" study from May 2021 to January 2022 (eight months) at the only COVID-19 vaccination center in Makokou, in Gabon’s Eastern Health Region. Volunteers were included during the vaccination process and responded to a questionnaire during a face-to-face interview. We studied their sociodemographic profile, knowledge of the disease and the vaccine, and perceptions and experiences of accessibility.

**Results:**

A total of 303 people between 18 and 71 years old, were included in the study. Subjects aged 30 to 40 years old represented 39.6% (n = 120) of those included. The sex ratio was 2.7. Eighty-six point seven percent of participants were of Gabonese nationality, and the median length of stay in the city of Makokou was six years (IQR: 2-15). The most represented professions were health workers (17.8%, n = 54), followed by defense and security forces (15.8%, n = 48), who were the main targets of the program. Forty-two point five percent (n = 129) of participants reported being influenced in their decision to get vaccinated. Globally, 72.6% (n = 220) thought that vaccination was a constraint, either for health reasons (n = 128, 58.2%) or for greater freedom of movement (n = 109, 49.5%). The main sources of information were the media (n = 237, 78.2%) and medical personnel (n = 168, 55.4%). Motivation was associated with compulsion (OR = 2.2), knowledge of certain symptoms (OR = 0.4), perception of the disease as serious (OR = 0.2), and positive media influence (OR = 0.4). Accessibility was associated with previous infection (OR = 11) or employer coercion (OR = 0.2).

**Conclusion:**

The decision to get vaccinated against COVID-19 was greatly influenced by the media and socio-professional constraints. Given that access to the vaccine was considered easy, motivation should have been the pursuit of better health.

## Introduction

Le premier cas de Covid-19, importé de Chine, a été identifié au Gabon le 12 mars 2020. Au vu du danger que représentait la Covid-19, le COPIL (Comité de pilotage du plan de veille et de riposte contre l’épidémie à coronavirus, au Gabon) a été mis en place et des mesures gouvernementales ont été prises pour limiter la propagation du virus dans le pays [20,22,28,33,36].

Au Gabon, du début de la pandémie jusqu’au 6 septembre 2023, 48 992 cas confirmés de Covid-19 ont été notifiés, avec 307 décès selon l’Organisation mondiale de la santé (OMS) [26]. La vaccination comme moyen de lutte et de riposte a été introduite par le COPIVAC (Comité de pilotage de la vaccination contre la Covid-19). En mars 2021, le Gabon a reçu 200 000 doses de vaccin Sinopharm® et a débuté sa première campagne de vaccination le 23 mars 2021. D’autres vaccins ont été disponibles par la suite, notamment les vaccins Pfizer® (10 620 doses en septembre 2021) et Johnson & Johnson® (168 000 doses en septembre 2021), mis à disposition par l’initiative COVAX (*COVID-19 Vaccines Global Access*) [15]. Ces vaccins ont été disponibles gratuitement pour les citoyens, d’abord à Libreville, puis progressivement dans les 113 centres fixes de vaccination des départements sanitaires pour couvrir le territoire. Le ministère de la Santé avait fixé comme objectif de vacciner 58 % de la population éligible d’ici la fin de l’année 2021. Les populations éligibles, identifiées à partir des recommandations de l’OMS, étaient : les personnels de santé civils et militaires; les agents des forces de défense et de sécurité; les personnes ayant des comorbidités; les agents des professions à risque élevé (enseignants, commerçants, etc.) et toute personne ayant un test positif à la Covid-19 datant de plus 3 mois. Malgré les efforts du ministère de la Santé et les campagnes médiatiques incitatives, l’objectif initial n’a pas pu être atteint : au mois de décembre 2022, seulement 13,6 % de la population avaient reçu au moins une dose d’un des vaccins disponibles et 11,3 % de la population étaient complètement vaccinés [36]. Ces faibles résultats au niveau national montraient que le Gabon n’échappait pas au climat de suspicion qui entourait la vaccination contre la Covid-19 [8,12,35].

Bien que la recommandation de vaccination apparaisse dans la quasi-totalité des notes de service et des discours des autorités administratives et politiques, elle n’a pas été déclarée officiellement obligatoire. Toutefois, les personnes non vaccinées étaient astreintes à présenter un test Covid-19 négatif datant de moins de 15 jours pour accéder à leur lieu de travail, dans certains espaces ouverts au public (restaurants, administrations, etc.) et pour effectuer les déplacements à l’intérieur du pays. Lors de l’introduction du vaccin, certaines mesures gouvernementales avaient été très incitatives, notamment l’allègement, pour les détenteurs d’une carte de vaccination complète, de l’obligation de faire les tests de dépistage lors des déplacements et des voyages à l’intérieur du pays, ou l’accès facilité à certains lieux publics, administrations, bars et restaurants.

Au Gabon, la propagation rapide de la pandémie de Covid-19 jusqu’aux endroits les plus éloignés, les mesures gouvernementales drastiques, l’introduction rapide d’une vaccination de masse avec l’exigence de la signature d’un consentement éclairé, ont été à l’origine d’un véritable raz-de-marée d’infodémie. Cette infodémie a créé doute et scepticisme sur l’existence de la maladie et l’intérêt de la vaccination. Ainsi des théories complotistes se sont propagées, *via* les réseaux sociaux, aboutissant à l’hésitation, voire au refus vaccinal dans certaines communautés comme cela a été observé dans d’autres pays [1,15,17,18,19, 22,23,25,30,34,35]. L’acceptation individuelle de la vaccination contre la Covid-19 n’a pas été aisée du fait que les États ne pouvaient garantir ni l’innocuité, ni l’efficacité du vaccin [1]. Néanmoins, une partie de la population avait tout de même accepté d’être vaccinée. Du fait d’un faible niveau d’adhésion au départ, il a fallu s’attendre à une faible couverture vaccinale, les motivations étant différentes d’un candidat à l’autre [6,31]. L’absence de données sur les motivations des candidats à la vaccination a suscité la réalisation de cette étude, dont l’objectif était d’évaluer les facteurs de motivation et la perception de l’accessibilité à la vaccination contre la Covid-19 dans la commune de Makokou.

## Patients et méthodes

Il s’agissait d’une étude observationnelle transversale à visée analytique sur les connaissances, attitudes et pratiques (CAP), basée sur un questionnaire préétabli (Annexe 1), réalisée au service de vaccination contre la Covid-19 du Centre hospitalier régional Omar Bongo Ondimba (CHROBOM) de Makokou, pendant les 8 premiers mois d’introduction du vaccin dans la région sanitaire, période allant de mai 2021 à janvier 2022.

La région sanitaire Est correspond à la région administrative de l’Ogooué-Ivindo [13,29]. Elle est divisée en 4 départements sanitaires et la densité de sa population est estimée à environ 2 habitants/km^2^. En 2020, les couvertures vaccinales de la plupart des antigènes du Programme élargi de vaccination (PEV), étaient les plus basses du pays. Le premier cas de Covid-19 dans la région a été déclaré le 13 juin 2020, soit trois mois après le premier cas national. Le premier centre de vaccination contre la Covid-19 dans cette région sanitaire a été ouvert au mois de mai 2021 au CHROBOM qui hébergeait déjà une unité de prise en charge des cas graves de Covid-19, au sein du service d’infectiologie. Ce centre de vaccination contre la Covid-19 recevait tous les candidats à la vaccination de la région sanitaire.

A la faveur de la campagne itinérante de vaccination mobile, d’autres centres ont ouvert dans les trois autres départements sanitaires de la région. Au 20 février 2022, l’Ogooué-Ivindo avait rapporté 312 cas confirmés de Covid-19, dont 7 décès. Le nombre de personnes complètement vaccinées, quel que soit le vaccin dans la région sanitaire durant la pandémie, était de 3 919, dont 3 286 à Makokou, soit environ 6 % de la cible régionale (données du COPIL).

Cette étude s’adressait à toute la population de la région Est éligible à la vaccination Covid-19. Lorsque le programme de vaccination contre la COVID-19 a été lancé, seul le centre de Makokou fonctionnait. Les centres de vaccination contre la COVID-19 des trois autres départements sanitaires de la région Est n’avaient pas encore été créés. Les critères d’éligibilité au vaccin avaient été définis comme suit par le COPIVAC :

- être âgé d’au moins 18 ans;
- donner son consentement éclairé;
- n’avoir pas fait de réaction grave à la première dose de vaccin;
- n’avoir pas fait d’accident vasculaire cérébral (AVC) au cours des trois derniers mois; ne pas présenter de saignements anormaux et des troubles de la coagulation;
- ne pas être enceinte et ne pas avoir planifié de l’être dans les prochains mois.

**Figure 1:**
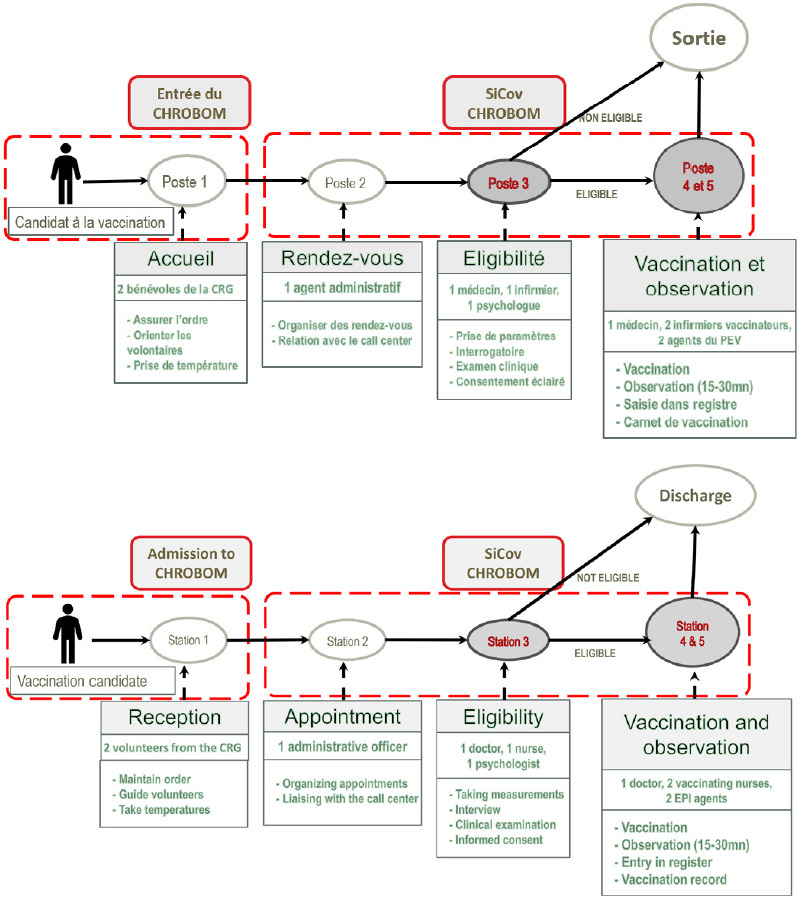
Étapes de la vaccination au CHROBOM en 2021. Le questionnaire était administré entre les postes 3 et 5. *CRG : Croix-Rouge Gabonaise

La vérification de ces critères se faisait à l’enrôlement, suivant un parcours de sélection décrit dans la Figure 1.

Nous avons inclus les sujets jugés éligibles à la vaccination, tenant compte des critères du COPIVAC, et ayant donné leur consentement pour la participation à l’étude.

Les critères de non-inclusion correspondaient aux critères de non éligibilité énoncés par le COPIVAC, notamment les sujets ayant fait un AVC au cours des trois derniers mois, ayant présenté des saignements anormaux ou des troubles de la coagulation, ainsi que les femmes ayant planifié une grossesse dans les prochains mois. Pour la collecte des données, il a été utilisé un questionnaire standardisé (annexe 1), conçu par le Département d’épidémiologie biostatistique et informatique médicale. Ce questionnaire comportait cinq parties qui concernaient respectivement : les caractéristiques sociodémographiques; les connaissances sur la maladie; les connaissances sur vaccination; les éléments de motivation; l’accessibilité à la vaccination.

L’étude était présentée au candidat, au début du circuit de vaccination mis en place par le COPIVAC (Fig. 1), pour recueillir son consentement. Une fiche de consentement libre et éclairé en double exemplaire était signée, un exemplaire pour le participant, l’autre pour les archives. Une fois le consentement signé, l’inclusion se faisait après la confirmation de l’éligibilité, au poste 3, où était réalisée une interview pour compléter le questionnaire de l’étude.

Les données collectées ont été saisies dans une base de données MS Excel®. Les variables âge, nombre d’enfants et durée de séjour dans la région ont été transformées en variables catégorielles à deux classes en prenant pour seuil leurs médianes respectives. La variable « catégorie socioprofessionnelle » a été transformée en une variable catégorielle à trois classes en regroupant les professions en trois cibles : la cible 1 contenait le personnel de santé et les personnels des forces de défense et de sécurité, la cible 2 les enseignants, les administrateurs des services publics et privés, les apprenants et la cible 3 les agriculteurs, les artisans, les commerçants, les ouvriers, les retraités, les sujets sans profession déclarée.

L’analyse de données a été réalisée avec le logiciel R version 4.2.2. Les variables qualitatives ont été décrites au moyen des effectifs et des proportions avec des intervalles de confiances à 95 % calculés par la méthode des intervalles de Wilson avec correction de continuité. Les variables quantitatives ont été décrites en utilisant la médiane et l’intervalle interquartile. Les proportions ont été comparées avec le test de Chi-2 de Mantel-Haenszel. Les facteurs associés à la motivation et à l’accessibilité ont été recherchés en réalisant une analyse de régression logistique, en utilisant la méthode pas à pas descendante. Les variables à expliquer étaient la motivation et l’accès à la vaccination Covid-19. Dans le modèle initial, nous avons pris en compte toutes les variables indépendantes ayant été associées à la variable dépendante au cours de l’analyse univariée. Le modèle de régression final a été obtenu en recherchant le résidu le plus faible possible. Les rapports de cote (OR) ont été enregistrés avec leurs intervalles de confiance correspondants à 95 % (IC à 95 %). Le seuil de significativité était fixé à 0,05.

## Résultats

De mai 2021 à janvier 2022, 1 769 personnes ont été vaccinées contre la Covid-19 au centre de vaccination de Makokou, parmi lesquelles 323 avaient accepté de participer à l’étude. Vingt questionnaires incomplets ont été retirés en raison de données manquantes. Ainsi, 303 personnes ont été incluses (Tableau I). Dans cette étude, la proportion des hommes était de 73 % (n = 220), le sex-ratio était de 2,71, l’âge médian des répondants était 36 ans (IIQ : 29-43). La proportion des vaccinés de nationalité gabonaise vivant à Makokou était de 86,7 % (n = 263). La médiane d’enfant par famille était de 3 (IIQ : 1-5). La durée médiane de séjour dans l’Ogooué-Ivindo était de 6 ans (IIQ : 2-15).

**Tableau I:** Répartition des 303 vaccinés contre la Covid-19 au CHROBOM à Makokou en 2021 selon leurs caractéristiques socio-démographiques

Caractéristique	Effectifs	Proportion (%)	IC95 %
Sexe			
masculin	220	73,0	[67,0 – 77,0]
féminin	83	27,0	[23,0 – 33,0]
Nationalité
gabonaise	263	86,7	[82,0 – 90,0]
camerounaise	11	3,6	[1,9 – 6,6]
chinoise	11	3,6	[1,9 – 6,6]
sénégalaise	4	1,3	[0,4 – 3,6]
autre	14	4,6	[2,6 – 7,8]
Ville de résidence
Makokou	248	82,0	[77,0 – 86,0]
autre	55	18,0	[14,0 – 23,0]
Religion
chrétienne	233	77,0	[72,0 – 81,0]
musulmane	35	11,5	[8,3 – 16,0]
autre	35	11,5	[8,3 – 16,0]
Niveau d’étude
supérieur	131	43,0	[38,0 – 49,0]
secondaire	159	52,0	[47,0 – 58,0}
primaire	8	2,6	[1,2 – 5,3]
non scolarisé	5	1,7	[0,6 – 4,0]
Nombre d’enfants
au plus 3 enfants	204	63,2	[58,0 – 68,0]
plus de 3 enfants	119	36,8	[32,0 – 42,0]
Catégorie socioprofessionnelle
cible 1	111	37,0	[31,0 – 42,0]
cible 2	101	33,0	[28,0 – 39,0]
cible 3	91	30,0	[25,0 – 36,0]

Les connaissances des enquêtés sur la maladie sont représentées dans le Tableau II, les connaissances sur le vaccin dans le Tableau III; la répartition selon la motivation de la vaccination est présentée dans le Tableau IV et celle selon la perception de l’accès à cette vaccination par le Tableau V. Le Tableau VI compare, en fonction du sexe, les connaissances des symptômes de la maladie et la source d’information; le Tableau VII explore le contact qu’ils ont eu avec la maladie, leurs niveaux d’inquiétude et d’exposition ressentis.

**Tableau II:** Répartition des 303 vaccinés contre la Covid-19 au CHROBOM à Makokou en 2021 selon les symptômes et signes de la maladie cités spontanément par eux

Caractéristique	Effectifs	Proportion (%)	IC95 %
Symptômes et signes cités spontanément			
fièvre	243	80,0	[75,0 – 85,0]
toux	228	75,0	[70,0 – 80,0]
problème respiratoire	188	62,0	[56,0 – 68,0]
mal de gorge	169	56,0	[50,0 – 62,0]
trouble du goût	165	55,0	[49,0 – 60,0]
trouble de l’odorat	159	53,0	[47,0 – 58,0]
douleur	129	43,0	[37,0 – 49,0]
écoulement nasal	127	42,0	[36,0 – 48,0]
céphalées	98	33,0	[27,0 – 38,0]
diarrhée	43	14,0	[11,0 – 19,0]
Sources d’où proviennent les connaissances sur la maladie			
média	237	78,2	[73,6 – 82,9]
personnel de santé	164	54,1	[48,5 – 59,7]
famille	46	15,2	[11,1 – 19,2]
entourage	38	12,5	[8,8 – 16,3]
formation	22	7,3	[4,3 – 10,2]

**Tableau III:** Répartition des 303 vaccinés contre la Covid-19 au CHROBOM à Makokou en 2021 selon leurs connaissances sur le vaccin

Caractéristique	Nombre de oui	Proportion (%)	IC95 %
Vaccination, meilleur moyen pour lutter contre la Covid-19	241	80,0	[74,0 – 84,0]
La vaccination contre la Covid-19 peut être évitée car :			
le vaccin n’a pas d’AMM	9	15,0	[7,3 – 26]
le vaccin n’est pas efficace	5	8,1	[3,0 – 19,0]
il y a peu de doses de vaccin	2	3,2	[0,5 – 12,0]
il y a trop de délai entre les doses	2	3,2	[0,5 – 12,0]
le vaccin est chinois, de mauvaise qualité	8	13,0	[6,1 – 24,0]
le vaccin a des effets secondaires	9	15,0	[7,3 – 26,0]
il existe d’autres moyens de prévention plus efficaces	10	16,0	[8,4 – 28,0]
autres raisons	7	5,6	[1,2 – 8,0]
Campagne de vaccination contre la Covid-19 en cours	263	89,0	[85,0 – 93,0]
Sources d’informations (combinées)
presse	115	38,0	[33,0 – 44,0]
radio	168	55,0	[50,0 – 61,0]
télévision	158	52,0	[46,0 – 58,0]
courrier	13	4,3	[2,4 – 7,4]
réunion	61	20,0	[16,0 – 25,0]
affichage	56	18,0	[14,0 – 23,0]
école	24	7,9	[5,2 – 12,0]
entourage	95	31,0	[26,0 – 37,0]
Appréciation de la couverture médiatique			
suffisante	139	45,9	[40,0 – 52,0]
pas d’avis	95	31,3	[26,0 – 37,0]
insuffisante	62	20,5	[16,0 – 26,0]
exaspérante	7	2,3	[1,0 – 4,9]
Perception du niveau d’information sur le vaccin			
oui	149	49,0	[43,0 – 55,0]
pas assez	110	36,0	[31,0 – 42,0]
non	44	15,0	[11,0 – 19,0]
Modification de l’opinion par la médiatisation	259	85,0	[81,0 – 89,0]
Influence de la médiatisation			
positive	217	84,0	[79,0 – 88,0]
négative	42	16,0	[12,0 – 21,0]

**Tableau IV:** Répartition des 303 vaccinés contre la Covid-19 au CHROBOM à Makokou en 2021 selon leurs motivations à se faire vacciner

Caractéristique	Nombre de oui	Proportion (%)	IC95 %
Idées de la vaccination avant la pandémie
n’est réservée qu’aux enfants	26	9,5	[6,4 – 14,0]
réservée aux personnes qui voyagent	74	27,0	[22,0 – 33,0]
réservée aux personnes malades	39	14,0	[10,0 – 19,0]
pour tout le monde quel que soit l’âge	186	68,0	[61,0 – 73,0]
réservée à certaines professions	21	7,6	[4,9 – 12,0]
Contexte vaccination Covid-19 différent des autres vaccinations	207	68,0	[63,0 – 73,0]
Incitation à la vaccination Covid-19 par cas grave déclaré dans le voisinage	232	77,0	[71,0 – 816,0]
Vaccination influencée par une personne ou facteur extérieur	129	43,0	[37,0 – 48,0]
Élément ayant influencé le choix vaccinal			
médecin	22	17,1	[11,0 – 25,0]
famille	36	27,9	[21,0 – 37,0]
employeur	27	20,9	[14,0 – 29,0]
chef d’établissement scolaire	6	4,7	[1,9 – 10,0]
exigence administrative	8	6,2	[2,9 – 12,0]
médiatisation	6	4,7	[1,9 – 10,0]
ami ou collègue	5	3,9	[1,4 – 9,3]
autre	35	27,1	[20,0 – 36,0]
Vaccination vue comme une contrainte	220	72,6	[67,0 – 77,0]
Raisons de contrainte combinées			
raisons de santé	128	58,2	[51,0 – 65,0]
plus de liberté	109	49,5	[43,0 – 56,0]
raisons professionnelles	61	27,7	[22,0 – 34,0]
effet de mode	4	1,8	[0,6 – 4,9]

**Tableau V:** Répartition des 303 vaccinés contre la Covid-19 au CHROBOM à Makokou en 2021 selon leur perception de l’accès à la vaccination

Caractéristique	Nombre de oui	Proportion (%)	IC95 %
Facilité d’accès au centre de vaccination	140	46,2	[41,0 – 52,0]
Raison de non accessibilité
distance	99	60,7	[53,0 – 68,0]
horaires	32	19,6	[14,0 – 27,0]
pas de transport	26	16,0	[11,0 – 23,0]
autre	6	3,0	[2,8 – 3,15]
Facilité d’accès au centre de vaccination	225	74,3	[69,0 – 79,0]
Facilité d’accès au centre de vaccination	166	54,8	[49,0 – 60,0]
Éléments les plus gênants combinés			
la longue attente	122	73,1	[66,0 – 79,0]
la longue procédure	68	40,7	[33,0 – 49,0]
le confort du centre	8	4,8	[2,2 – 9,5]
l’humeur du personnel	8	4,8	[2,2 – 9,5]
Organisation du centre de vaccination			
bonne	176	58,1	[52,0 – 64,0]
moyenne	120	39,6	[34,0 – 45,0]
mauvaise	7	2,3	[1,0 – 4,9]

**Tableau VI:** Répartition des vaccinés contre la Covid-19 au CHROBOM à Makokou en 2021 selon leurs connaissances sur la maladie (principaux symptômes cités spontanément)

Caractéristique	Féminin*	Masculin*	Total	p-valeur**
Fièvre	72 (29,6)	171 (70,4)	243 (100,0)	0,049
Toux	73 (32,0)	155 (68,0)	228 (100,0	<0,001
Respiration	56 (29,8)	132 (70,2)	188 (100,0)	0,2
Gorge	52 (30,8)	117 (69,2)	169 (100,0)	0,11
Goût	45 (27,3)	120 (72,7)	165 (100,0)	>0,9
Odorat	49 (30,8)	110 (69,2)	159 (100,0)	0,13
Douleur	36 (27,9)	93 (72,1)	129 (100,0)	0,8
Écoulement nasal	34 (26,8)	93 (73,2)	127 (100,0)	0,9
Céphalées	28 (28,6)	70 (71,4)	98 (100,0)	0,7
Diarrhée	16 (37,2)	27 (62,8)	43 (100,0)	0,11
Principales sources de connaissances de la maladie				
personnel de santé	44 (26,8)	120 (73,2)	164 (100,0)	0,4
famille (causerie)	9 (19,6)	37 (80,4)	46 (100,0)	0,14
formation (travail)	8 (36,4)	14 (63,6)	22 (100,0)	0,4
média	65 (27,4)	172 (72,6)	237 (100,0)	0,3
entourage	13 (34,2)	25 (65,8)	38 (100,0)	0,4

* n(%) **test du khi-deux d’indépendance

**Tableau VII:** Répartition des vaccinés contre la Covid-19 au CHROBOM à Makokou en 2021, selon le contact qu’ils ont eu avec la maladie, leurs niveaux d’inquiétude, d’exposition ressentie, en fonction du sexe

Caractéristique	Féminin*	Masculin*	Total	p**
Contracté la Covid-19	7 (43,8)	9 (56,3)	16 (100,0)	0,2
Dépisté pour la Covid-19	43 (24,9)	130 (75,1)	173 (100,0)	0,3
Malade Covid-19 dans l’entourage immédiat	26 (25,2)	77 (74,8)	103 (100,0)	0,5
Peur de la Covid-19	66 (26,4)	184 (73,6)	250 (100,0)	0,4
Raisons de la peur				
maladie grave	80 (28,2)	204 (71,8)	284 (100,0)	0,4
maladie mortelle	76 (28,3)	193 (71,7)	269 (100,0)	0,5
maladie avec séquelles	11 (36,7)	19 (63,3)	30 (100,0)	0,2
maladie fréquente	4 (36,4)	7 (63,6)	11 (100,0)	0,5
maladie médiatisée	7 (28,0)	18 (72,0)	25 (100,0)	>0,9
Échelle d’inquiétude				0,2
0	1 (100,0)	0 (0,0)	1 (100,0)	
1	3 (16,7)	15 (83,3)	18 (100,0)	
2	14 (32,6)	29 (67,4)	43 (100,0)	
3	18 (34,6)	34 (65,4)	52 (100,0)	
4	21 (30,4)	48 (69,6)	69 (100,0)	
5	26 (21,8)	93 (78,2)	119 (100,0)	
6	0 (0,0)	1 (100,0)	1 (100,0)	
Exposition supérieure aux autres personnes	28 (23,5)	91 (76,5)	119 (100,0)	0,15

* n(%) **test exact de Fisher; test du khicarré d’indépendance

**Figure 2:**
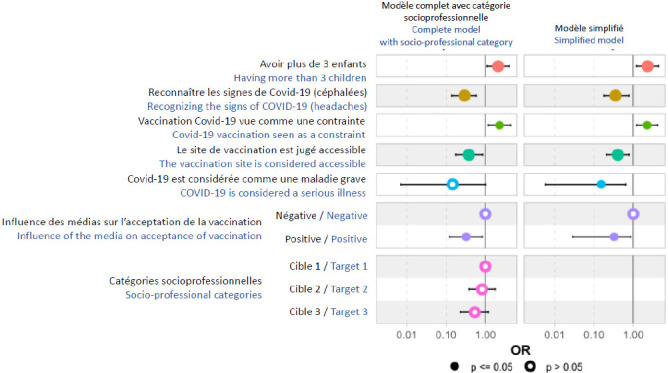
Rapport de cote et intervalle de confiance des facteurs associés à la motivation de se faire vacciner chez les candidats à la vaccination Covid-19 en 2021 au CHROBOM à Makokou (modèle final)

Les résultats de l’analyse multivariée sont exposés dans le Tableau VIII. La catégorie socioprofessionnelle, dont nous avons forcé l’introduction dans le modèle final, n’a pas eu d’influence majeure (Fig. 2) sur les autres variables du modèle.

**Tableau VIII:** Rapport de cote et intervalle de confiance des facteurs associés à la motivation de se faire vacciner chez les candidats à la vaccination Covid-19 en 2021 au CHROBOM à Makokou (modèle final multivarié)

Caractéristique	Rapport de cote	95 % IC	p-valeur**
Avoir plus de 3 enfants	2,6	[1,5 – 4,6]	<0,001
Reconnaître les signes de Covid-19 (Céphalées)	0,4	[0,2 – 0,8]	0,008
Vaccination Covid vue comme une contrainte	2,2	[1,1 – 4,2]	0,021
Le site de vaccination est jugé accessible	0,5	[0,3 – 0,8]	0,008
Covid-19 considérée comme une maladie grave	0,2	[0,02 – 0,6]	0,014
Influence des médias sur l’acceptation de la vaccination			
négative	1,0	–	
positive	0,4	[0,2 – 0,9]	0,030

**Figure 3:**
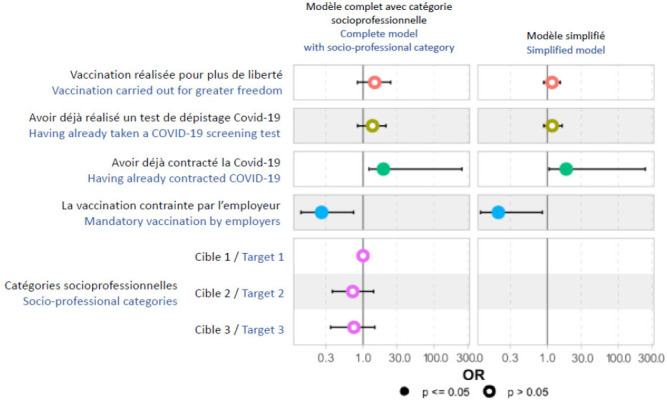
Rapport de cote et intervalle de confiance des facteurs associés à l’accessibilité physique de la vaccination chez les candidats à la vaccination Covid-19 en 2021 au CHROBOM à Makokou (modèle final)

Finalement, quatre variables ont été retenues comme associées à l’accessibilité : avoir déjà contracté la Covid-19, avoir déjà réalisé un test de dépistage Covid-19, avoir été contraint à la vaccination par l’employeur, et vouloir être vacciné pour plus de liberté (Tableau IX). De même que pour la motivation, la catégorie socioprofessionnelle, que nous avons introduite dans le modèle final, n’a pas eu d’influence majeure sur les autres variables du modèle (Fig. 3).

**Tableau IX:** Rapport de cote et intervalle de confiance des facteurs associés à la motivation de se faire vacciner chez les candidats à la vaccination Covid-19 en 2021 au CHROBOM à Makokou (modèle final multivarié)

Caractéristique	Rapport de cote	95 % IC	p-valeur**
Avoir déjà contracté la Covid-19	11,4	[1,5 – 248]	0,041
Avoir déjà réalisé un test de dépistage Covid-19	2,1	[0,8 – 5,6]	0,13
La vaccination contrainte par employeur	0,2	[0,02 – 0,7]	0,028
La vaccination réalisée pour plus de liberté	2,0	[0,8 – 5,0]	0,2

## Discussion

L’objectif de cette étude était d’évaluer les facteurs de la motivation et de l’accessibilité à la vaccination contre la Covid-19 dans la commune de Makokou. L’échantillonnage de notre enquête n’est pas représentatif de la population de la région, car prélevé au sein d’une structure sanitaire. Le but était de faire une esquisse des raisons pour lesquelles ces personnes avaient accepté la vaccination, du fait de toutes les influences extérieures, comme l’infodémie, autour de cette vaccination. Une étude en population générale, incluant les personnes vaccinées ou non, aurait eu du mal à être réalisée en situation de confinement et de distanciation physique, comme une étude récente réalisée au Cameroun, durant la même période [4].

Dans notre étude près de 3/4 des répondants avaient un âge compris entre 29 et 43 ans, ce qui correspond à une population jeune. Cette observation était comparable à celles qui avaient été rapportées dans d’autres études des pays de l’Afrique centrale, notamment au Cameroun [11] et en République démocratique du Congo [7]. Les répondants avaient majoritairement fait des études supérieures (43 %), en proportion plus importante que celle rapportée par une étude congolaise [7], mais inférieure au résultat d’une étude camerounaise [11]. Cette disparité pourrait s’expliquer par la différence des tailles des échantillons des différentes études mais aussi par les contextes sociaux différents, le taux de scolarisation étant variable d’un pays à l’autre.

Les principales sources d’information étaient les médias (65 %) et le personnel de santé (44 %), retrouvées également dans d’autres études africaines [3]. L’influence positive des médias (84 %) dans l’acquisition des connaissances et la motivation à se faire vacciner, très marquée dans notre étude, a été également observée en Tanzanie [16] et au Nigeria [10]. Toutefois, en fonction du contexte social, la médiatisation de cette maladie peut avoir des répercussions négatives et même contreproductives comme cela a été observé en Afrique du Sud [5]. Cela suggère qu’une combinaison de méthodes et de stratégies de sensibilisation, numériques et traditionnelles, peut être nécessaire pour répondre de manière globale à la réticence à la vaccination, comme cela a été montré à Madagascar [27].

Les motivations de la population de Makokou ne concernaient pas la confiance dans les soins de santé, le désir de protéger leurs amis et leur famille ou des considérations de santé personnelles, contrairement à ce qu’avaient observé Elbers et Makadzange [9,21].

La vaccination contre la Covid-19 n’a pas connu une grande popularité contrairement aux vaccinations du PEV de routine, plus ancré dans les mentalités et les traditions depuis 1974. Dans les deux cas, une meilleure communication et des explications sur les effets secondaires sont toujours nécessaires pour améliorer l’adhésion et continuer à soutenir la lutte contre les maladies évitables par la vaccination, afin d’éviter les forts taux de refus, comme observés au Cameroun [11] par exemple. Une étude réalisée en Afrique de l’Ouest, au Niger, nous semble décrire les mêmes réalités que celles observées au Gabon [32].

Divers facteurs socioculturels ont amené les populations à percevoir la vaccination contre la Covid-19 comme une contrainte en Afrique, notamment les inégalités sociales de santé historiques, le passé colonial, la désinformation et les croyances culturelles [2]. L’un des arguments évoqués par les antivaccins était qu’il s’agissait d’une expérimentation à laquelle les Africains participaient à leur insu; un autre argument était qu’il s’agissait d’une maladie inventée; un autre que la pharmacopée traditionnelle africaine était suffisamment efficace pour traiter cette « petite grippe ». Les effets indésirables réels ou supposés du vaccin amenaient également à l’hésitation, voire au refus [6,14]. Ce qui a encouragé les remèdes traditionnels, qui dans notre contexte africain sont assez nombreux pour traiter une simple grippe [37,24]. La contrainte socioprofessionnelle vient également enrichir les facteurs socioculturels. Dans un contexte où l’amélioration des couvertures vaccinales est un perpétuel défi, ces éléments pourraient avoir un impact sur l’acceptation et l’utilisation des vaccins contre les maladies à prévention vaccinale courantes à travers le continent.

## Conclusion

Dans notre étude, la motivation pour la vaccination contre la Covid-19 était essentiellement due à une contrainte professionnelle, clairement définie à l’embauche ou subtilement imposée par d’autres exigences, rendant cette vaccination quasi obligatoire. La localisation excentrée du centre de vaccination et le manque de transports urbains sont apparus comme un facteur limitant l’accès à cette vaccination. La décision de se faire vacciner a été beaucoup influencée par les médias et les contraintes socioprofessionnelles, alors que la motivation aurait dû être la recherche d’un meilleur état de santé plutôt qu’une contrainte plus ou moins voilée.

## Comité d’éthique, consentement du patient ou autorisation administrative

Cette étude avait obtenu l’autorisation du ministère de la Santé à travers la Direction générale de la santé (DRS) de l’Ogooué Ivindo. Cette autorisation a été jointe. L’interrogation des sujets était assujettie à l’obtention préalable de leur consentement après leur avoir expliqué l’objet de cette enquête. Un exemplaire de consentement signé par un répondant a été joint.

## Remerciements

Remerciements à toutes les personnes qui ont accepté de répondre au questionnaire et aux psychologues du CHROBOM qui ont aidé à réaliser cette étude.

## Financement

Aucun.

## Contributions des auteurs

Élaboration du protocole et analyse des données :

Bisvigou, Minko, Ibinga

Recueil des données : Minko

Rédaction du manuscrit : Bisvigou, Ibinga

Relecture du manuscrit : Maghendji Nzondo, Ibinga

Coordination de la recherche : Ngoungou

## Déclaration de liens d’intérêt

Aucun lien d’intérêt n’a été déclaré.

## Figures and Tables

**Figure:**
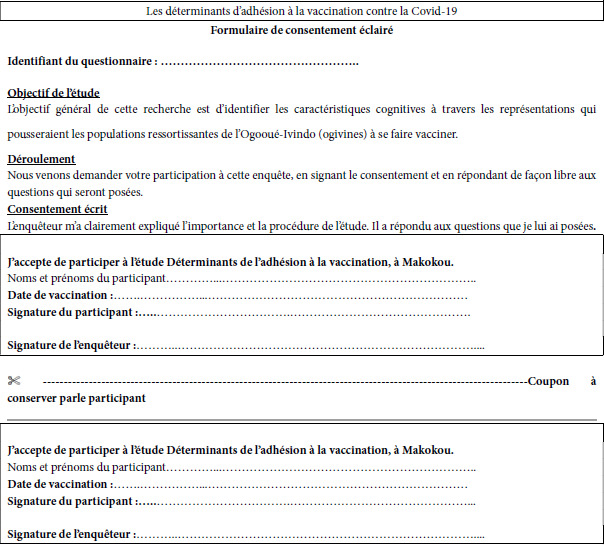


**Figure:**
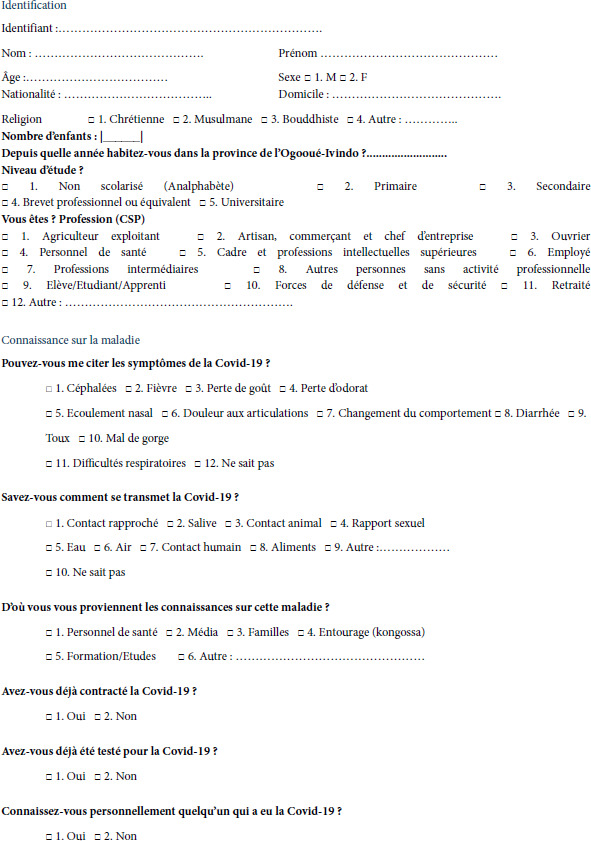


**Figure:**
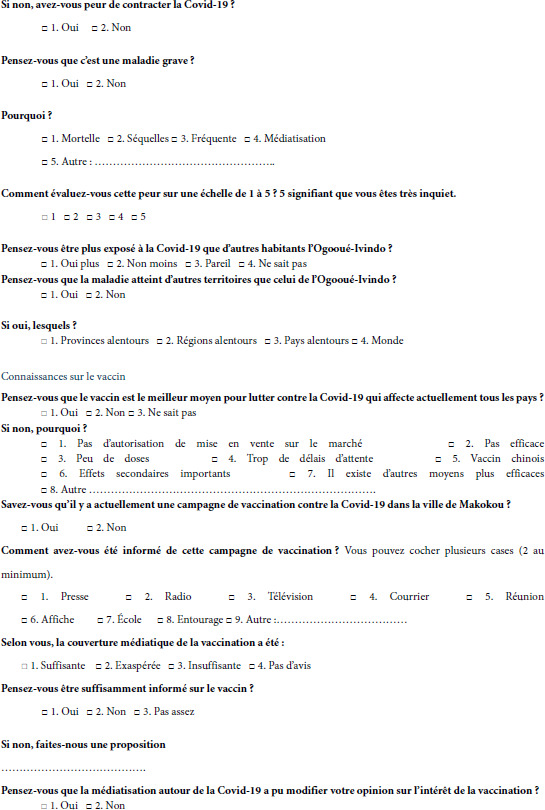


**Figure:**