# Supplementary material for: Motivation and perception of vaccine accessibility among people vaccinated against COVID-19 in Makokou, Gabon, in 2021–2022
Source: Med Trop Sante Int. 2026 Jan 29;6(1):mtsi.v6i1.2026.514. [Article in French] doi: 10.48327/mtsi.v6i1.2026.514 (PMC13229047; doi:10.48327/mtsi.v6i1.2026.514)
Supplement: Related texts

# Research article: Motivation and perception of vaccine accessibility among people vaccinated against COVID-19 in Makokou, Gabon, in 2021–2022

## Introduction

The first case of imported COVID-19 was identified in Gabon on March 12, 2020. Due to the danger posed by the virus, the COPIL (the Steering Committee for the Coronavirus Epidemic Monitoring and Response Plan in Gabon) was established, and the government took measures to limit its spread in the country [20,22,28,33,36]. From the start of the pandemic until September 6, 2023, Gabon reported 48,992 confirmed cases of COVID-19, resulting in 307 deaths, according to the World Health Organization (WHO) [26]. The COPIVAC (Steering Committee for the Coronavirus Vaccination) introduced vaccination as a means of control and response. In March 2021, Gabon received 200,000 doses of the Sinopharm® vaccine and began its first vaccination campaign on March 23, 2021. Other vaccines subsequently became available, including Pfizer® (10,620 doses in September 2021) and Johnson & Johnson® (168,000 doses in September 2021) vaccines. These vaccines were made available through the COVAX (COVID-19 Vaccines Global Access) Initiative [15]. These vaccines were made available free of charge to citizens first in Libreville and then gradually expanded to 113 fixed vaccination centers in health departments across the country. The Ministry of Health set a goal to vaccinate 58% of the eligible population by the end of 2021. The eligible populations, as defined by WHO recommendations, included civilian and military health workers, defense and security forces personnel, individuals with comorbidities, and workers in high-risk professions (e.g., teachers and shopkeepers). Additionally, those who had tested positive for COVID-19 more than three months prior were eligible.

Despite the Ministry of Health’s efforts and media campaigns encouraging vaccination, the initial target was not met. By December 2022, only 13.6% of the population had received at least one dose of one of the available vaccines, and 11.3% of the population was fully vaccinated [36]. These poor national-level results showed that Gabon was not immune to the climate of suspicion surrounding the COVID-19 vaccine [8,12,35].

Although the recommendation to get vaccinated appeared in nearly all memos and speeches by administrative and political authorities, vaccination was not officially declared mandatory. However, unvaccinated individuals were required to present a negative COVID-19 test result from within the last 15 days to access their workplace and certain public spaces, such as restaurants and government offices. They were also required to present such a result to travel within the country. When the vaccine was introduced, the government implemented several encouraging measures, including relaxing the requirement for individuals with full vaccination cards to undergo screening tests when traveling within the country and providing easier access to certain public places, government offices, bars, and restaurants.

In Gabon, the rapid spread of the pandemic to even the most remote areas, drastic government measures, and the rapid introduction of mass vaccination requiring an informed consent form, caused a tidal wave of misinformation. This in-fodemic has created doubt and skepticism about the existence of the disease and the benefits of vaccination. Consequently, conspiracy theories spread via social media, leading to hesitancy and refusal to vaccinate in certain communities, as observed in other countries [1,15,17,18,19,22,23, 25,30,34,35]. Acceptance of the vaccine has not been easy for individuals, as governments could not guarantee its safety or efficacy [1]. Nevertheless, some of the population agreed to be vaccinated. Due to the low initial uptake, low vaccination coverage was expected, as motivations varied from candidate to candidate [6,31]. The lack of data on the motivations of vaccination candidates prompted this study, which aimed to assess the motivating factors and perceptions of accessibility to the vaccine in the municipality of Makokou.

## Patients and methods

This cross-sectional, observational study focused analytically on knowledge, attitudes, and practices (KAP) and was based on a pre-established questionnaire (see Appendix 1). The study was conducted at the Omar Bongo Ondimba Regional Hospital Center (CHROBOM)’s COVID-19 vaccination service in Makokou from May 2021 to January 2022, during the first eight months of the vaccine’s introduction in the health region. The Eastern Health Region corresponds to the Ogooué-Ivindo administrative region [13,29]. It is divided into four health departments and has an estimated population density of approximately two inhabitants per square kilometer. In 2020, the region had the lowest vaccination coverage for most antigens in the Expanded Program on Immunization (EPI) in the country. The first case of COVID-19 infection in the region was reported on June 13, 2020, three months after the first national case. The first vaccination center for this health region opened in May 2021 at CHROBOM. The center was located within the infectious diseases department, which already housed a unit for treating severe cases of the virus. This center received all vaccination candidates from the health region.

Thanks to the mobile vaccination campaign, other centers opened in the three other health departments in the region.

As of February 20, 2022, Ogooué-Ivindo had reported 312 confirmed cases of COVID-19, including seven deaths. During the pandemic, the number of people fully vaccinated, regardless of the vaccine, in the health region was 3,919, including 3,286 in Makokou, which is about 6% of the regional target (COPIL data).

This study targeted the entire Eastern region population eligible for the vaccine. When the COVID-19 vaccination program was launched, only the Makokou center was in operation. The COVID-19 vaccination centers in the three other health departments of the East region had not yet been set up.

The COPIVAC eligibility criteria for the vaccine are as follows:

- Be at least 18 years of age.
- Give informed consent.
- Have not had a serious reaction to the first dose of the vaccine.
- Have not had a stroke in the last three months.
- Have no abnormal bleeding or coagulation disorders.
- Not be pregnant or planning to become pregnant in the coming months.

These criteria were verified at enrollment, following the selection process described in Figure 1. Subjects who were deemed eligible for vaccination according to the COPIVAC criteria and who gave their consent to participate in the study were included.

**Figure 1:** CHROBOM’s vaccination steps in 2021. Questionnaire was administered between posts 3 and 5. *CRG: Gabonese Red Cross volunteers

Subjects were excluded if they met the COPIVAC ineligibility criteria, which included having suffered a stroke in the previous three months, having abnormal bleeding or coagulation disorders, or being pregnant or planning to become pregnant in the coming months.

We used a standardized questionnaire (Appendix 1), designed by the Department of Epidemiology, Biostatistics, and Medical Informatics, to collect data. The questionnaire had five sections covering sociodemographic characteristics, knowledge about the disease, knowledge about vaccination, motivational factors, and accessibility to vaccination.

At the start of the vaccination program set up by COPIVAC (Fig. 11), the study was presented to the candidate to obtain their consent. A free and informed consent form was signed in duplicate, with one copy given to the participant and one copy kept in the archives. After signing the consent form, participants were included in the study after confirming their eligibility at station 3, where they were interviewed to complete the study questionnaire.

The collected data were entered into an Excel® database. Age, number of children, and length of stay in the region were transformed into two-class categorical variables using their respective medians as thresholds. The “socio-professional category” variable was transformed into a three-class categorical variable by grouping professions into three categories. Target 1 included health workers and defense and security personnel; target 2 included teachers, public and private service administrators, and students; and target 3 included farmers, artisans, merchants, workers, retirees, and individuals with no declared occupation.

Data analysis was performed using R software, version 4.2.2. Qualitative variables were described using frequencies and proportions, and 95% confidence intervals were calculated using the Wilson interval method with continuity correction. Quantitative variables were described using the median and interquartile range. Proportions were compared using the Mantel-Haenszel chisquare test. Logistic regression analysis using the stepwise descent method was performed to investigate factors associated with motivation and accessibility. The dependent variables were motivation and access to the vaccination. In the initial model, we included all independent variables associated with the dependent variables in the univariate analysis. The final regression model was obtained by seeking the lowest possible residual. We recorded odds ratios (OR) with their corresponding 95% confidence intervals (95% CI). The significance threshold was set at 0.05.

## Results

From May 2021 to January 2022, 1,769 people received the COVID-19 vaccine at the Makokou vaccination center. Of those individuals, 323 agreed to participate in the study. Twenty incomplete questionnaires were excluded due to missing data. Thus, 303 people were included (Table I). The proportion of men in this study was 73% (n = 220), the sex ratio was 2.71, and the median age of respondents was 36 years (interquartile range [IQR]: 29–43). Eighty-six point seven percent (n = 263) of Gabonese nationals living in Makokou had been vaccinated. The median number of children per family was three (interquartile range [IIQ]: one to five). The median length of stay in the Ogooué-Ivindo region was six years (interquartile range [IIQ]: two to 15 years).

**Table I:** Distribution of Covid-19 vaccinated at the CHROBOM in Makokou in 2021 by socio-demographic characteristics

Characteristic	Participants	Proportion (%)	IC95 %
Gender			
male	220	73,0	[67,0 – 77,0]
female	83	27,0	[23,0 – 33,0]
Nationality			
Gabonese	263	86,7	[82,0 – 90,0]
Cameroonian	11	3,6	[1,9 – 6,6]
Chinese	11	3,6	[1,9 – 6,6]
Senegalese	4	1,3	[0,4 – 3,6]
other	14	4,6	[2,6 – 7,8]
City of residence			
Makokou	248	82,0	[77,0 – 86,0]
other	55	18,0	[14,0 – 23,0]
Religion			
Christian	233	77,0	[72,0 – 81,0]
Muslim	35	11,5	[8,3 – 16,0]
other	35	11,5	[8,3 – 16,0]
Level of education			
higher education	131	43,0	[38,0 – 49,0]
secondary education	159	52,0	[47,0 – 58,0}
primary education	8	2,6	[1,2 – 5,3]
no schooling	5	1,7	[0,6 – 4,0]
Number of children			
up to 3 children	204	63,2	[58,0 – 68,0]
more than 3 children	119	36,8	[32,0 – 42,0]
Socio-professional category			
target 1	111	37,0	[31,0 – 42,0]
target 2	101	33,0	[28,0 – 39,0]
target 3	91	30,0	[25,0 – 36,0]

Table II shows the respondents’ knowledge of the disease, Table III shows their knowledge of the vaccine, Table IV shows the distribution according to motivation for vaccination, and Table V shows the distribution according to perceived access to vaccination. Table VI compares knowledge of the disease’s symptoms and sources of information by gender. Table VII explores contact with the disease, levels of concern, and perceived exposure. The results of the multivariate analysis are shown in Table VIII. The socio-professional category, which we deliberately included in the final model, did not have a major influence (Fig. 2).

**Figure 2:** Odd ratio and confidence interval of factors associated with motivation to be vaccinated among 2021 COVID-19 candidates at the CHROBOM in Makokou (final model)

**Table II:** Distribution of Covid-19 vaccinated at the CHROBOM in Makokou in 2021 by symptoms and signs of the disease mentioned spontaneously by them

Characteristic	Participants	Proportion (%)	95% CI
Symptoms and signs reported spontaneously			
fever	243	80,0	[75,0 – 85,0]
cough	228	75,0	[70,0 – 80,0]
breathing difficulties	188	62,0	[56,0 – 68,0]
sore throat	169	56,0	[50,0 – 62,0]
taste disturbance	165	55,0	[49,0 – 60,0]
smell disturbance	159	53,0	[47,0 – 58,0]
pain	129	43,0	[37,0 – 49,0]
runny nose	127	42,0	[36,0 – 48,0]
headaches	98	33,0	[27,0 – 38,0]
diarrhea	43	14,0	[11,0 – 19,0]
Sources of knowledge about the disease			
media	237	78,2	[73,6 – 82,9]
healthcare personnel	164	54,1	[48,5 – 59,7]
family	46	15,2	[11,1 – 19,2]
friends and relatives	38	12,5	[8,8 – 16,3]
training	22	7,3	[4,3 – 10,2]

**Table III:** Distribution of the 303 people vaccinated against COVID-19 at CHROBOM in Makokou in 2021 according to their knowledge of the vaccine

Characteristic	Number of yes votes	Proportion (%)	95% CI
Vaccination, the best way to fight COVID-19	241	80,0	[74,0 – 84,0]
Vaccination against COVID-19 can be avoided because:			
the vaccine does not have marketing authorization	9	15,0	[7,3 – 26]
the vaccine is not effective	5	8,1	[3,0 – 19,0]
there are few doses of vaccine available	2	3,2	[0,5 – 12,0]
there is too long a delay between doses	2	3,2	[0,5 – 12,0]
the vaccine is Chinese and of poor quality	8	13,0	[6,1 – 24,0]
the vaccine has side effects	9	15,0	[7,3 – 26,0]
there are other, more effective means of prevention	10	16,0	[8,4 – 28,0]
other reasons	7	5,6	[1,2 – 8,0]
Covid-19 vaccination campaign underway	263	89,0	[85,0 – 93,0]
Sources of information (combined)			
press	115	38,0	[33,0 – 44,0]
radio	168	55,0	[50,0 – 61,0]
television	158	52,0	[46,0 – 58,0]
mail	13	4,3	[2,4 – 7,4]
meeting	61	20,0	[16,0 – 25,0]
posters	56	18,0	[14,0 – 23,0]
school	24	7,9	[5,2 – 12,0]
friends and relatives	95	31,0	[26,0 – 37,0]
Assessment of media coverage			
sufficient	139	45,9	[40,0 – 52,0]
no opinion	95	31,3	[26,0 – 37,0]
insufficient	62	20,5	[16,0 – 26,0]
exasperating	7	2,3	[1,0 – 4,9]
Perception of the level of information about the vaccine			
yes	149	49,0	[43,0 – 55,0]
not enough	110	36,0	[31,0 – 42,0]
no	44	15,0	[11,0 – 19,0]
Change of opinion through media coverage	259	85,0	[81,0 – 89,0]
Influence of media coverage
positive	217	84,0	[79,0 – 88,0]
negative	42	16,0	[12,0 – 21,0]

**Table IV:** Distribution of the 303 people vaccinated against COVID-19 at CHROBOM in Makokou in 2021 according to their reasons for getting vaccinated

Characteristic	Number of yes votes	Proportion (%)	95% CI
Ideas about vaccination before the pandemic			
only for children	26	9,5	[6,4 – 14,0]
only for people who travel	74	27,0	[22,0 – 33,0]
reserved for sick people	39	14,0	[10,0 – 19,0]
for everyone regardless of age	186	68,0	[61,0 – 73,0]
reserved for certain professions	21	7,6	[4,9 – 12,0]
Covid-19 vaccination context differs from other vaccinations	207	68,0	[63,0 – 73,0]
Encouragement to get the Covid-19 vaccine due to a serious case reported in the neighborhood	232	77,0	[71,0 – 816,0]
Vaccination influenced by an external person or factor	129	43,0	[37,0 – 48,0]
Factor influencing vaccine choice			
doctor	22	17,1	[11,0 – 25,0]
family	36	27,9	[21,0 – 37,0]
employer	27	20,9	[14,0 – 29,0]
school principal	6	4,7	[1,9 – 10,0]
administrative requirement	8	6,2	[2,9 – 12,0]
media coverage	6	4,7	[1,9 – 10,0]
friend or colleague	5	3,9	[1,4 – 9,3]
other	35	27,1	[20,0 – 36,0]
Vaccination seen as a constraint	220	72,6	[67,0 – 77,0]
Combined reasons for constraint			
health reasons	128	58,2	[51,0 – 65,0]
more freedom	109	49,5	[43,0 – 56,0]
professional reasons	61	27,7	[22,0 – 34,0]
fashion trend	4	1,8	[0,6 – 4,9]

**Table V:** Distribution of the 303 people vaccinated against COVID-19 at CHROBOM in Makokou in 2021 according to their perception of access to vaccination

Characteristic	Number of yes votes	Proportion (%)	95% CI
Ease of access to the vaccination center	140	46,2	[41,0 – 52,0]
Reason for inaccessibility			
distance	99	60,7	[53,0 – 68,0]
timetables	32	19,6	[14,0 – 27,0]
no transportation	26	16,0	[11,0 – 23,0]
other	6	3,0	[2,8 – 3,15]
Ease of access to the vaccination center	225	74,3	[69,0 – 79,0]
Ease of access to the vaccination center	166	54,8	[49,0 – 60,0]
Most annoying factors combined			
the long wait	122	73,1	[66,0 – 79,0]
the lengthy procedure	68	40,7	[33,0 – 49,0]
the comfort of the center	8	4,8	[2,2 – 9,5]
the mood of the staff	8	4,8	[2,2 – 9,5]
Organization of the vaccination center			
good	176	58,1	[52,0 – 64,0]
avearge	120	39,6	[34,0 – 45,0]
poor	7	2,3	[1,0 – 4,9]

**Table VI:** Distribution of people vaccinated against COVID-19 at CHROBOM in Makokou in 2021 according to their knowledge of the disease (main symptoms cited spontaneously)

Characteristic	Female*	Male*	Total	p-value**
Fever	72 (29,6)	171 (70,4)	243 (100,0)	0,049
Cough	73 (32,0)	155 (68,0)	228 (100,0	<0,001
Breathing	56 (29,8)	132 (70,2)	188 (100,0)	0,2
Throat	52 (30,8)	117 (69,2)	169 (100,0)	0,11
Taste	45 (27,3)	120 (72,7)	165 (100,0)	>0,9
Smell	49 (30,8)	110 (69,2)	159 (100,0)	0,13
Pain	36 (27,9)	93 (72,1)	129 (100,0)	0,8
Runny nose	34 (26,8)	93 (73,2)	127 (100,0)	0,9
Headaches	28 (28,6)	70 (71,4)	98 (100,0)	0,7
Diarrhea	16 (37,2)	27 (62,8)	43 (100,0)	0,11
Main sources of knowledge about the disease				
healthcare personnel	44 (26,8)	120 (73,2)	164 (100,0)	0,4
family (chat)	9 (19,6)	37 (80,4)	46 (100,0)	0,14
training (work)	8 (36,4)	14 (63,6)	22 (100,0)	0,4
media	65 (27,4)	172 (72,6)	237 (100,0)	0,3
friends and relatives	13 (34,2)	25 (65,8)	38 (100,0)	0,4

* n(%) **chi-square test of independence

**Table VII:** Distribution of COVID-19 vaccinated at the CHROBOM in Makokou in 2021, according to their contact with the disease, their levels of concern, exposure felt, by sex

Characteristic	Female*	Male*	Total	p**
Contracted COVID-19	7 (43,8)	9 (56,3)	16 (100,0)	0,2
Tested for COVID-19	43 (24,9)	130 (75,1)	173 (100,0)	0,3
Someone close to me has COVID-19	26 (25,2)	77 (74,8)	103 (100,0)	0,5
Fear of COVID-19	66 (26,4)	184 (73,6)	250 (100,0)	0,4
Reasons for fear				
serious illness	80 (28,2)	204 (71,8)	284 (100,0)	0,4
life-threatening illness	76 (28,3)	193 (71,7)	269 (100,0)	0,5
illness with lasting effects	11 (36,7)	19 (63,3)	30 (100,0)	0,2
common illness	4 (36,4)	7 (63,6)	11 (100,0)	0,5
illness covered by the media	7 (28,0)	18 (72,0)	25 (100,0)	>0,9
Anxiety scale				0,2
0	1 (100,0)	0 (0,0)	1 (100,0)	
1	3 (16,7)	15 (83,3)	18 (100,0)	
2	14 (32,6)	29 (67,4)	43 (100,0)	
3	18 (34,6)	34 (65,4)	52 (100,0)	
4	21 (30,4)	48 (69,6)	69 (100,0)	
5	26 (21,8)	93 (78,2)	119 (100,0)	
6	0 (0,0)	1 (100,0)	1 (100,0)	
Higher exposure than others	28 (23,5)	91 (76,5)	119 (100,0)	0,15

* n(%) **Fisher’s exact test; chi-square test of independence

**Table VIII:** Odd ratio and Confidence interval of factors associated with motivation to be vaccinated among 2021 COVID-19 candidates at the CHROBOM in Makokou (final multivariate model)

Characteristic	Odd ratio	95% CI	p-value**
Having more than 3 children	2,6	[1,5 – 4,6]	<0,001
Recognizing the signs of COVID-19 (headaches)	0,4	[0,2 – 0,8]	0,008
COVID vaccination seen as a constraint	2,2	[1,1 – 4,2]	0,021
The vaccination site is considered accessible	0,5	[0,3 – 0,8]	0,008
COVID-19 considered a serious disease	0,2	[0,02 – 0,6]	0,014
Media influence on vaccination acceptance			
negative	1,0	–	
positive	0,4	[0,2 – 0,9]	0,030

Four variables were identified as being associated with accessibility: having previously contracted or tested positive for COVID-19, being forced by an employer to receive the vaccine, and wanting to be vaccinated for greater freedom (Table IX). As with motivation, the socio-professional category did not have a significant impact on the other variables in the final model (Fig. 33).

**Figure 3:** Odd ratio and confidence interval of factors associated with access among 2021 COVID-19 candidates at the CHROBOM in Makokou (final model)

**Table IX:** Odd ratio and Interval of confident of factors associated with access to immunization among 2021 COVID-19 candidates at the CHROBOM in Makokou (initial model)

Characteristic	Odd ratio	95% CI	p-value**
Having already contracted COVID-19	11,4	[1,5 – 248]	0,041
Having already taken a COVID-19 test	2,1	[0,8 – 5,6]	0,13
Vaccination required by employer	0,2	[0,02 – 0,7]	0,028
Vaccination for greater freedom	2,0	[0,8 – 5,0]	0,2

## Discussion

This study aimed to evaluate the factors influencing motivation and accessibility to the COVID-19 vaccine in the municipality of Makokou.

However, the sample for our survey is not representative of the region’s population because it was taken from a healthcare facility. We aimed to outline the reasons why these individuals agreed to be vaccinated, despite the external influences surrounding this vaccination, such as the infodemic. Studying the general population, including both vaccinated and unvaccinated individuals, would have been difficult in a lockdown situation with physical distancing, as in a recent study conducted in Cameroon during the same period [4].

In our study, nearly three-quarters of the respondents were between 29 and 43 years old, indicating a young population. This finding is consistent with those reported in other studies in Central African countries, particularly Cameroon [11] and the Democratic Republic of Congo [7]. Most respondents had a higher level of education (43%), a higher proportion than in a Congolese study [7] but lower than in a Cameroonian study [11]. This disparity could be explained by differences in sample sizes and social contexts, as well as varying school enrollment rates from one country to another.

The main sources of information were the media (65%) and health personnel (44%), as found in other African studies [3]. The positive influence of the media on acquiring knowledge and motivation to get vaccinated was very pronounced in our study and has also been observed in Tanzania [16] and Nigeria [10]. However, depending on the social context, media coverage of this disease can have negative, even counterproductive, repercussions, as was observed in South Africa [5]. These findings suggest that a combination of digital and traditional awareness-raising methods and strategies is necessary to comprehensively address vaccine hesitancy, as demonstrated in Madagascar [27]. Contrary to what Elbers and Makadzange had observed [9,21], the motivations of the Makokou population did not relate to trust in healthcare, the desire to protect friends and family, or personal health considerations.

Unlike routine EPI vaccinations, which have been deeply rooted in mindsets and traditions since 1974, the acceptance of the COVID-19 vaccine has not been widespread. Better communication and explanations about side effects are needed in both cases to improve uptake and continue supporting the fight against vaccine-preventable diseases and avoid high refusal rates, as observed in Cameroon, for example. A study conducted in West Africa, in Niger, seems to describe realities similar to those observed in Gabon [32].

Various sociocultural factors have led populations in Africa to perceive the vaccine as a constraint, including historical social inequalities in health, colonial history, misinformation, and cultural beliefs [2]. Anti-vaccine advocates have argued that Africans are unwitting participants in an experiment, that the disease was invented, and that traditional African medicine is effective enough to treat this “minor flu.” The real or supposed side effects of the vaccine have also led to hesitation or refusal [6,14]. This has encouraged the use of traditional remedies, of which there are many in the African context for treating a simple flu [37,24]. Socio-professional constraints add to these socio-cultural factors. In a context where improving vaccination coverage is a constant challenge, these factors could impact the acceptance and use of vaccines against common, vaccine-preventable diseases across the continent.

## Conclusion

In our study, the motivation for receiving the COVID-19 vaccine was primarily due to professional constraints. These constraints were either clearly defined at the time of hiring or subtly imposed by other requirements, effectively making the vaccine mandatory. The remote location of the vaccination center and the lack of urban transportation appeared to limit access to the vaccination. The decision to get vaccinated was greatly influenced by the media and socio-professional constraints. Ideally, the motivation would be the pursuit of better health rather than more or less veiled constraints.

## Ethics committee, patient consent, or administrative authorization

This study was authorized by the Ministry of Health through the General Directorate of Health (DRS) of Ogooué Ivindo. This authorization is attached. Subjects were interviewed only after providing their consent and after the purpose of the survey had been explained to them. A copy of the signed consent form is attached.

## Acknowledgments

We would like to thank all of the respondents who agreed to complete the questionnaire, as well as the psychologists at CHROBOM who helped carry out this study.

## Funding

This study did not receive any funding.

## Authors’ contributions

Protocol development and data analysis: Bisvigou, Minko, and Ibinga.

Data collection: Minko

Manuscript writing: Bisvigou and Ibinga

Manuscript review: Maghendji Nzondo and Ibinga

Research Coordination: Ngoungou

## Conflict of Interest

The authors declare no conflicts of interest.

## Appendix: Questionnaire
